# Exploration of Weld Bead Forming Rule during Double-Pulsed GMAW Process Based on Grey Relational Analysis

**DOI:** 10.3390/ma12223662

**Published:** 2019-11-07

**Authors:** Ping Yao, Kang Zhou, Hongyan Lin, Zihui Xu, Songchen Yue

**Affiliations:** 1 College of Electromechanical Engineering, Guangdong Polytechnic Normal University, Guangzhou 510635, China; gsyaop@gpnu.edu.cn (P.Y.); sjy843644862@163.com (H.L.); xzh110592@163.com (Z.X.); 2 School of Mechatronical Engineering, Beijing Institute of Technology, Beijing 100081, China; Yue_Songchen@163.com

**Keywords:** DP-GMAW process, grey relational analysis, twin pulse relation, average welding current

## Abstract

Weld bead forming rule is very important during double-pulsed gas metal arc welding (DP-GMAW) process, and this process has more advantages than that of conventional arc welding process. This work employed grey rational analysis to explore the weld bead forming rule. Since the latest *twinpulse* XT DP control process was employed, the parameters adjustment was easier than that of conventional operation. The grey relational analyses between five main process parameters, which were average welding current, welding speed, twin pulse relation, twin pulse frequency together with twin pulse current change in percent, and three key characteristic parameters, which were bead width, bead height and penetration, were conducted to explore the weld bead forming rule. To accurately calculate the grey relational degree, the negative relevancies were transformed to positive ones. According to calculations and corresponding analyses, it can be concluded that the effects of average welding current and welding speed on the weld bead forming and key characteristic parameters of the weld bead were higher than that of other process parameters. Moreover, the relevancies between key characteristic parameters of the weld bead, and process parameters which included twin pulse relation, average welding current and twin pulse current change in percent were positive, while the relevancies between key characteristic parameters and other two process parameters were negative. The work can supply a new method to evaluate the effects of process parameters during the DP-GMAW process on the weld bead forming or other process characteristics, and references for parameters selection and process optimization.

## 1. Introduction

As an effective metallic material joining method, double-pulsed gas metal arc welding (DP-GMAW) process has been employed in many industrial areas [1]. This technique was an improvement based on conventional the pulsed GMAW (P-GMAW) process, which has been investigated and reported since 1950s [2]. DP-GMAW process refers to more advantages when compared to the P-GMAW process, such as DP-GMAW process has wider welding current range to adjust, can be applied for all position welding and wider joint gap configuration, also, double pulses can reduce overall heat delivery and improve the joint property, and strongly stir the weld pool and reduce porosity and crack sensitivity [3,4]. Hence, it has been used more and more in actual industrial occasions. 

Welding quality evaluation or estimation is very important for almost all the relative operations [5,6]. The product of the DP-GMAW process is weld bead, so the welding quality evaluation is to estimate the weld bead using various special criteria. The geometry condition is one the most important and obvious characteristics for evaluating the quality, due to this is an intuitive condition which can be closely related to the mechanical properties, distortion of the welded joint, and deposition efficiency [7], not only in DP-GMAW process, but also in various arc welding processes whose outputs are weld bead. The geometry information includes the height, penetration and width of the bead, which are closely related to the heat delivery, absorption and phase change of the base plates. 

During the DP-GMAW process, there are various process operational parameters related to the forming of the weld bead. How to explore the effects of these parameters on the weld bead, or relate the operational process parameters to the geometry of the weld bead in various arc welding processes has been seriously considered by a lot of scholars and experts. Many relative important contributions were published during past decades. Shen et al. [8] investigated the effects of heat delivery on the weld bead geometry, percent dilution and melting efficiency using different heat input for four plates during submerged arc welding process. For GMAW process, Rodrigues et al. [9] studied the influences of shielding gas (argon, argon-hydrogen and argon-helium mixtures) and two activating fluxes (a commercial flux and a TiO_2_ based flux) on the geometry of weld beads produced by TIG (tungsten inert gas, one type GMAW process) arc welding process. Also, Shoeb et al. [10] studied the effects of the process parameters, including welding speed, voltage and gas flow rate on the bead geometry, and established relative mathematical equations to present the relations using factorial form. These relative works demonstrated significance of the geometry of weld bead in welding relative researches. 

In addition, there are various process parameters during the DP-GMAW process, and the effects of different process parameters on the forming of the weld bead are so different, therefore, the effects of these parameters on the quality of welding products are also different. To design proper control method with appropriate process parameters combination, the effects should be seriously explored in detail. Also, as for the geometry of the weld bead, there are some characteristic parameters requiring to be analyzed, such as the bead width, bead height or penetration. Hence, the DP-GMAW operation system in this work should be a typical multi-input-multi-out (MIMO) system. 

To obtain weld beads with satisfactory quality, appropriate control methods should be conducted. However, the relations between key process parameters and characteristic parameters of weld bead should be clearly obtained. To explore the weld bead forming rule, a lot of experts employed various methods and many achievements have been made recently. Palani et al. [11] employed three key operational parameters, which were welding current, welding speed and nozzle-to-plate distance to design a three factor, five level central composite experimental program, in order to establish a second-order polynomial regression mathematical model to predict the weld bead geometry conditions, which included the penetration, bead width, reinforcement (bead height) and dilution, during the cladding by flux cored arc welding process. The same polynomial regression forming can also be conducted by Sen et al. [7]. Though the works can obtain the relations, the methods involved lots of process parameters and many coefficients required to be confirmed. Apart from using polynomial equation in the works mentioned before, advance artificial intelligent (AI) methods were also employed to obtain reliable results. Ghanty et al. [12] employed two types of neural networks, which were respectively multilayer perceptron (MLP) and radial basis function (RBF) neural networks, to map four important features, which were welding current, voltage, vertex angle and torch speed, into the bead width, penetration depth and bead area, and 45 experiments were conducted to collect the training data. They also used the same methods to predict weld bead geometry using features derived from the infrared thermal video [13]. Similar work was also conducted by Nagesh et al. [14]. Wang et al. [15] used types of back-propagation (BP) neural networks to relate the selected process parameters and geometry parameters. The works included eleven input process parameters but only two output parameters. Also, fuzzy rule-based model [16] and genetic algorithm [17] were employed to predict or optimize the geometry of the weld bead. Though the published works can obtain corresponding some relations, they lacked deep interpretation of the weld bead forming rule, especially the influential levels of the different key operational parameters on the key characteristic parameters of weld bead based on theoretical and experimental analyses. Also, majority of the conclusions of the works were based on several experiments instead of detailed characteristics analysis, hence, there should be a large improvement required in this research area. 

To explore the influential levels of different process parameters on the characteristic parameters of the weld bead, grey relational analysis, which is a powerful tool to assess quantitative and qualitative relationship between factors and variables using a relatively small amount of data [18], was employed in this work. This analysis method can identify the influential level of objects to be identified on the research objects through comparing the different relational degrees, under the circumstance that the number of specimens is so limited. This method had been widely employed in various areas, such as in assessing sustainable performance of energy systems [19,20], assessing influential factors of heat transfer in a blast furnace hearth [21], optimization of surface textured journal bearing [22], and relative other areas. In this work, this method was employed to analyze the influential factors of different process parameters on the characteristic parameters of weld bead, and then induced corresponding important rule about weld bead forming during the DP-GMAW process. 

In this work, the grey relational analysis was employed to achieve a goal that obtaining the influential levels of various process parameters on the characteristic parameters of weld bead by means of calculating the grey relational degrees, under the circumstance that limited amount of experiments were conducted. We hope the work can support and instruct the selection and combination of various process parameters, decrease the number of process experiments required, and supply a research foundation of setting proper process parameters and controlling the weld bead forming process. The rest of this paper is organized as follows: Section 2 will provide the principle of the DP-GMAW process based on robot operation. Section 3 will introduce the experimental platform and detailed designing program. Section 4 will about the experimental results presentation, analyses and discussion. Finally, Section 5 will provide some concluding remarks and suggestions for future work. 

## 2. Principle and Characteristics of the DP-GMAW Process 

**Figure 1:**
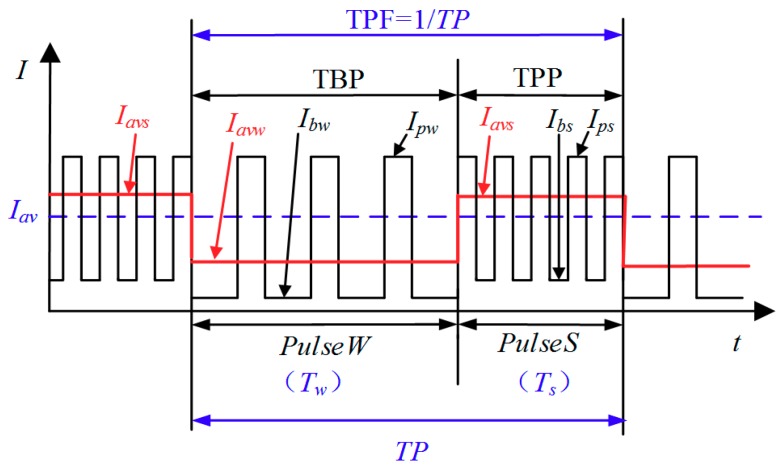
Schematic of the current waveform of the DP-GMAW process.

DP-GMAW process refers to a high-frequency current pulse waveform that was modulated by a low-frequency current pulse, which is called as thermal pulse [23]. The role of the high-frequency current pulse is to control the droplet transfer behavior to obtain a proper welding penetration, while the main function of the low-frequency current pulse is to obtain a series of regular pulses to stir the weld pool [24]. These two types of current pulses have enough benefits for refining grains and improving the quality of the weld joint [25]. During the process, according to the difference of the current waveforms, there are two distinct phases, which are thermal pulse phase (TPP) and thermal base phase (TBP), having different frequencies. The current waveforms in TPP has higher switching frequency than that in TBP. Hence, the current pulses in TPP is called as strong pulse set, and can be marked as *PulseS*; while the current pulses in TBP is called as weak pulse set, and can be marked as *PulseW*. The durations of these two pulse sets are respectively marked as *T_s_* and *T_w_*, and the sum of *T_w_* and *T_s_* can be considered as one thermal period, which is marked as *TP*. Correspondingly, the reciprocal of the *TP* can be considered as the frequency of the DP-GMAW operation, in this work we use TPF to describe this frequency. In general, TPF is used to reflect the varying speed of the pulse sets in TPP and TBP phases, and each set may include up to 10–20 high frequency pulses, whose maximum frequency may achieve 100 Hz. Hence, under the common circumstance, the value of the TPF during the process may below 5 Hz. Figure 1 showed the schematic of the current waveform of this process. It can be noticed that apart from some parameters which has been mentioned before, there are some current parameters during the process. The current peak value and base value in TPP phase are respectively marked as *I_ps_* and *I_bs_*, and the average current in this phase is *I_avs_*; while for the current in TBP phase, corresponding marks are *I_pw_*, *I_bw_* and *I_avw_*. Moreover, the average welding current of the whole process can be marked as *I_av_*. 

In addition, during the process, there are some other process parameters which are important for process controls and analyses. The first is the twin pulse current change, whose value is half of the difference between *I_avs_* and *I_avw_*. To clearly reflect the current adjustment, this parameter can be described combing the *I_av_* and using the percent format, corresponding mathematical description can be as follows:

(1) $$I_{\Delta }=\frac{I_{avs}-I_{avw}}{2I_{av}}\times 100\%$$

where *I*_Δ_ is named as twin pulse current change in percent. Moreover, the proportion of the time of TPP in one thermal period, can be denoted as twin pulse relation during the process, corresponding mathematical description is as follows:

*D_T_* = *T_s_*/*TP* (2)

where *D_T_* is used to denote the twin pulse relation. 

According to above illustrations, there are so many process parameters included during the DP-GMAW operational process, which make the analyzing process difficult and constructive conclusions cannot be easily drawn, because so many process parameters can increase the burden of the calculations and analyses, and the uncertainties of the results. In this work, to obtain clear and accurate relations between operational process parameters and characteristic parameters of the weld bead, a latest advanced control technology, which was *twinpulse* XT DP control process developed by LORCH Company (Lorch Schweißtechnik GmbH, Im Anwänder, Auenwald, Germany), was employed to decrease the number of process parameters which required to be adjusted during the operation process. There are two remarkable advantages in employing this control process. The first is that the frequencies of the current pulses in TPP and TBP are higher than those in the traditional DP-GMAW process, in general, the frequencies can respectively achieve 100 Hz and 30 Hz for the current pulses in TPP and TBP. The second is that when the average welding current in one thermal period remains constant, the peak and base values of the current pulses in the two phases are unchanged. Hence, the currents adjustment can be conducted only by means of changing the average welding current *I_av_* and the twin pulse relation *D_T_*, other various process parameters are not required to be considered during the heat delivery process. Therefore, the process control can be more effective and convenient when this control process was employed, also, the exploration of the weld bead forming rule can be more accurate and reliable. 

## 3. Experimental Design of the DP-GMAW Based on Robot Operation

To explore the rule of the weld bead forming during the DP-GMAW process, actual experiments should be seriously designed and conducted. To achieve optimum control performance and obtain accurate and reliable experimental and measured data, this work employed an industrial robot to assist the welding operation and control. In general, the arc welding based on industrial robot operation can accurately control the welding speed, in order to assure during the same welding time, the welding torch goes through the base plate with the same length. In addition, the welding torch can maintain a constant inclination, and other auxiliary instruments can be effectively employed to collect and analyze the process data during the process. In a word, employing an industrial robot to assist the DP-GMAW process can not only increase the production efficiency, but also improve the accuracy of the control operation. These features can benefit the objective of the research in this work. 

### 3.1. Experimental Platform and Materials 

**Figure 2:**
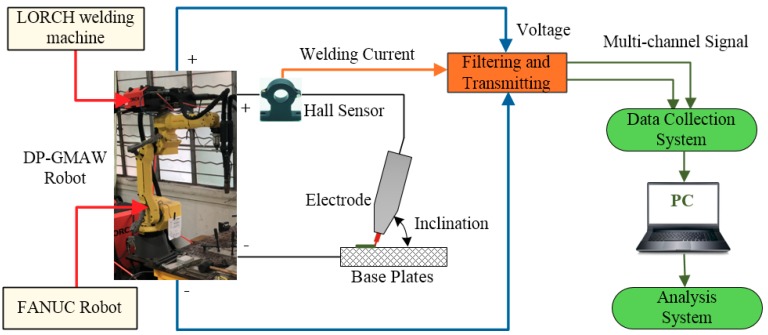
Experimental platform used in this work.

The experimental platform used in this work was composed of a FANUC Robot M-10IA industrial robot (FANUS Corporation, Oshino-mura, Yamanashi Prefecture, Japan), LORCH S-RobotMIG arc welding machine (Lorch Schweißtechnik GmbH, Im Anwänder, Auenwald, Germany) and other auxiliary equipment. The industrial robot controlled the welding speed, and the inclination of the electrode, while the current waveforms in TPP and TBP were controlled by the LORCH arc welding machine. In addition, one self-designed robot welding multi-signals collection and analysis instrument, which was based on the USB-6363 multi-channel signal acquisition card developed by NI (National Instruments) company (Austin, TA, USA), was utilized to synchronously collect the current, voltage, arc sound signals, and then the output signals can be transmitted into a computer for further calculation and analysis. Figure 2 shows the schematic of the experimental platform design. 

During the process, the base plate used stainless steel 304, whose tensile strength was 520 MPa; while the welding wire used stainless steel 316 L with 1.2 mm of diameter. The shielding gas was composed of 98% pure argon and 2% CO_2_ (15 L/min flow), the length of stick-out was 12 mm, and flat surfacing welding was used. The size of the base plate was 250 mm × 100 mm × 3 mm. The material characteristics of the base plate and the welding wire was shown in Table 1.

### 3.2. Experimental Program

In this work, *twinpulse* XT DP control process was employed. Under the circumstance, the number of adjustable parameters was so less. To obtain the effects of main process parameters on the weld bead forming rule during the DP-GMAW process, five process parameters in this new control process, which were twin pulse relation *D_T_*, welding speed *V_R_*, twin pulse frequency TPF, twin pulse current change in percent *I_∆_* and average welding current *I_av_*, were selected to explore the effects. According to our previous works about this process [26], the reasonable varying ranges of these five parameters had been confirmed. Then for each process parameter, different values were employed. To achieve comprehensive and further exploration, total 26 experiments which corresponded different process parameters combinations were designed and conducted in this work, which can be shown in Table 2. To clearly present the variation of each process parameter in the table, different color backgrounds were employed to highlight the corresponding variations of the five process parameters. It can be noticed that apart from the data which was highlighted using different color background, other data for one process parameter was the same for different experiments. 

During the experimental process, to assure obtaining reliable and accurate experimental and observation results, each array of the experiments was conducted three times, and then the results of the most stable experiments were chosen to do further analyses. 

### 3.3. Experimental Data Processing

To accurately obtain key experimental data and then explore the rule of the weld bead forming, each base plate used in the experiment should be carefully preprocessed. In this work, the surface of the base plate had been processed by angle grinder to eliminate the oxides, and then seriously washed by special alcohol. Then, after the surface of base plate clear and dry enough, welding actions can be taken. 

**Figure 3:**
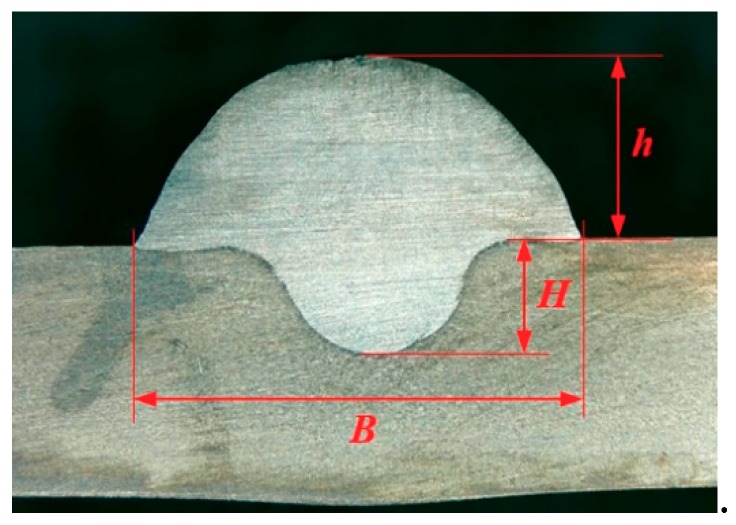
Weld bead geometry and parameters definitions.

To explore the rule of weld bead forming, some key characteristic parameters should be collected for each experiment. The parameters can be chosen according to the appearance of the weld bead. Figure 3 showed an actual photo and detailed characteristic parameters definitions of a weld bead. In the Figure 3, three characteristic parameters were marked, they were respectively bead width *B*, bead height *h*, and penetration *H*. To obtain accurate data of these parameters, these three values were independently measured by two staffs. Firstly, for *B* and *h*, five positions were selected in the middle of the weld bead, then using Vernier caliper and electron microscope to independently measure. For total four collected values for each parameter, the maximum and minimum values were rejected, and then a mean value could be considered as a final measurement value. This method can avoid random errors appearing during the measurement. As for the penetration *H*, serious sampling measurement should also be conducted. Two weld bead specimens with 1.5 cm of length in the middle of the weld bead were incised, and then the specimens were polished, cleaned by anhydrous alcohol and metallographic corroded. After the specimens was dried, clear appearance of the cross section of the weld bead can be obtained. Similarly, two methods, which respectively employed Vernier caliper and electron microscope, were used and then more data had been collected. Then the mean value of each measurement was considered as a final value of the penetration *H*. Hence, the accuracy of the measurement in this work can be guaranteed and convinced. 

In this work, grey system theory and corresponding grey relational analysis method was used to process the experimental data. Grey system theory was established by Professor Deng in 1982 [27], who proposed a new method to focus on the problems with less data and limited information. The grey relational analysis was a multi-factor statistical analysis method in the grey system theory. Its analysis was conducted for the grey control system using relational method. This method did not require a large number of data, so it can avoid data loss resulted from asymmetrical information. It can confirm the effects of process parameters on the operation results when the number of specimens were not enough and the relations between the elements and the results were not clear. In this work, the weld bead forming during the DP-GMAW process was affected by twin pulse relation, welding speed, twin pulse frequency and other process parameters. However, which process parameters had higher or lower effects, and what corresponding rules were, were required to be further focused on or researched. Hence, this work employed the grey relational analysis method to analyze the effects of different process parameters on the weld bead forming. The work can serve the further rule research about the welding process, instruct the parameter selection and predict key information of weld bead forming. 

The analysis can be executed by following procedures. 

*Step* 1: Confirm the data of the analysis element sequence based on analysis objective, which was the three key characteristic parameters of the weld bead forming in this work, and then collected the analysis data.

Set *n* data in the analysis element sequence and constitute a matrix as follows:

(3) $$(X_{1}^{'},X_{2}^{'},\cdots ,X_{n}^{'})=\left[\begin{array}{ccccc} x_{1}^{'}\left(1\right) & x_{2}^{'}\left(1\right) & x_{3}^{'}\left(1\right) & \cdots & x_{n}^{'}\left(1\right)\\ x_{1}^{'}\left(2\right) & x_{2}^{'}\left(2\right) & x_{3}^{'}\left(2\right) & \cdots & x_{n}^{'}\left(2\right)\\ x_{1}^{'}\left(3\right) & x_{2}^{'}\left(3\right) & x_{3}^{'}\left(2\right) & \cdots & x_{n}^{'}\left(3\right)\\ \vdots & \vdots & \vdots & \vdots & \vdots \\ x_{1}^{'}\left(m\right) & x_{2}^{'}\left(m\right) & x_{3}^{'}\left(m\right) & \cdots & x_{n}^{'}\left(m\right) \end{array}\right]$$

where *m* is the number of the element, and $X_{i}^{'}={\left(x_{i}^{'}\left(1\right),x_{i}^{'}\left(2\right),\cdots ,x_{i}^{'}\left(m\right)\right)}^{T}\;,i=1,2,\cdots ,n$ In this work, the value of *n* was 5. In other words, there were five data in the analysis element sequence: *X*_1_′ denoted the twin pulse relation, *X*_2_′ denoted the traveling speed of the industrial robot, *X*_3_′ denoted the twin pulse frequency, *X*_4_′ denoted the current change in percent and *X*_5_′ denoted the average welding current. Since the number of parameters combinations was 26, the value of *m* was 26. Hence, the total above data can compose of an *m* × *n* matrix. 

*Step* 2: Confirm the data in the reference sequence

The reference sequence should be an ideal comparing criterion, the data in the reference sequence can choose the optimum or the worst values, or choose other values based on special objectives.

(4) $$X_{0}^{'}={\left(x_{0}^{'}(1),x_{0}^{'}(2),\cdots x_{0}^{'}(m)\right)}^{T}$$

In this work, the characteristic parameters, which were bead width *B*, bead height *h* and penetration *H*, were chosen as the data which constituted the reference sequence, and then the researches can focus on the relational degree between these three key characteristic parameters and above five process parameters in analysis element sequence. 

*Step* 3: Perform normalization operations on the data of each element 

Because each data in the analysis element sequence had different physical meanings and had different units. To conveniently compare the data, the data can be normalized by mean value. The detailed algorithm was as follows:

(5) $$x_{i}\left(k\right)=\frac{x_{i}^{'}\left(k\right)}{\frac{1}{m}\sum_{k=1}^{m}x_{i}^{'}\left(k\right)}$$

Then the matrix of the data in the analysis element sequence (Equation (3)) can be transformed as follows:

(6) $$\left(X_{0},X_{1},\cdots ,X_{n}\right)=\left[\begin{array}{cccc} x_{0}\left(1\right) & x_{1}\left(1\right) & \cdots & x_{n}\left(1\right)\\ x_{0}\left(2\right) & x_{1}\left(2\right) & \cdots & x_{n}\left(2\right)\\ \vdots & \vdots & \vdots & \vdots \\ x_{0}\left(m\right) & x_{1}\left(m\right) & \cdots & x_{n}\left(m\right) \end{array}\right]$$

*Step* 4: Calculate the absolute error between each value in the data included in the analysis element sequence and corresponding data in the reference sequence, the calculation was as follows:

(7) $$\left|x_{0}\left(k\right)-x_{i}\left(k\right)\right|,\;\mathrm{where}\;k=1,\;2\ldots m,\;I=1,\;2\ldots .n$$

*Step* 5: (1). Confirm the minimum value:

(8) $$min_{i=1}^{n}min_{k=1}^{m}\left|x_{0}\left(k\right)-x_{i}\left(k\right)\right|$$

(2). Confirm the maximum value:

(9) $$max_{i=1}^{n}max_{k=1}^{m}\left|x_{0}\left(k\right)-x_{i}\left(k\right)\right|$$

*Step* 6: Calculate the relational coefficient sequence, corresponding equation was as follows:

(10) $$\zeta_{i}\left(k\right)=\frac{min_{i=1}^{n}min_{k=1}^{m}\left|x_{0}\left(k\right)-x_{i}\left(k\right)\right|+\rho \cdot max_{i=1}^{n}max_{k=1}^{m}\left|x_{0}\left(k\right)-x_{i}\left(k\right)\right|}{\left|x_{0}\left(k\right)-x_{i}\left(k\right)\right|+\rho \cdot max_{i=1}^{n}max_{k=1}^{m}\left|x_{0}\left(k\right)-x_{i}\left(k\right)\right|}$$

where *ρ* was the distinguishing coefficient, and the value range was between 0 and 1. Smaller value of *ρ* meant that the difference between relation coefficients was larger and the distinguishing ability was stronger. In general, the value of *ρ* was 0.5, which also was adopted by this work. 

*Step* 7: Calculate the grey relational degree

For each element, which should be evaluated the effect on the data in the reference, calculated the mean value of the corresponding relational coefficients in order to reflect the relational relation between data in the analysis element sequence and corresponding data in the reference sequences, this value was named as grey relational degree, the detailed calculation was as follows:

(11) $$r_{0i}=\frac{1}{m}\overset{m}{\underset{k=1}{\sum }}\zeta_{i}\left(k\right)$$

*Step* 8: Obtain the final grey relational rank for each analysis result according to the corresponding grey relational degrees. 

## 4. Experimental Results and Analyses

### 4.1. Appearance of the Weld Bead and Size

The experiments from R1 to R7 were to explore the effect of twin pulse relation *D_T_* on the weld bead forming. In these 7 experiments, the *D_T_* was increased from 20% to 80% with 10% of interval, at the same time other parameters were unchanged. The weld bead appearances and cross sections of the specimens R1–R7 were shown in Table 3. It can be noticed that as increasing of the *D_T_*, the forming of weld beads in specimens R1–R7 were satisfied, and the welding process were also stable. In addition, the bead widths from specimens R1–R7 were increasing with limited magnitudes. Also, according to the cross sections of the weld beads, it can be noticed that as increasing of the twin pulse relation, the melting area was increasing, and the bead width *B* together with bead height *h* also had significant increases. On the other hand, the changes of penetration *H* were not obvious. 

Though some varying rules can be obtained according to directly observe the appearances and measurement values, the rules were so limited. In addition, all the 26 specimens which were to explore all the selected five process parameters were seriously measured, and corresponding measurements were showed in Table 4. 

### 4.2. Grey Relational Analysis for Single Process Objective

#### 4.2.1. The Influential Rules of Process Parameters on Bead Width and Corresponding Relational Degree

**Figure 4:**
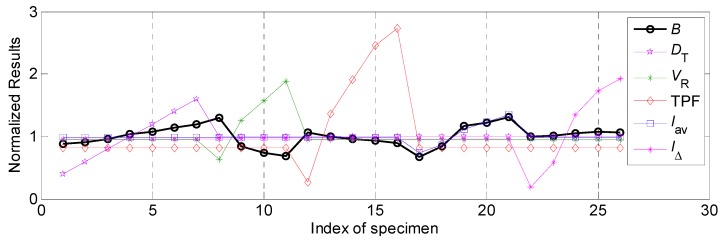
The normalized results of various process parameters and bead width.

In this work, the effects of various process parameters on the bead width *B* was first focused on. According to the *Step* 3 in Section 3.3, the normalized results of the bead width *B* and various process parameters, such as twin pulse relation *D_T_*, welding speed *V_R_*, twin pulse frequency TFP, average welding current *I_av_* and twin pulse current change in percent *I*_∆_, were shown in Figure 4. According to this presentation, the relational rule between various process parameters and bead width can be explored. The explorations about different process parameters should combine the Table 2 in Section 3.2. It can be noticed from Figure 4 that the relevance between twin pulse relation *D_T_* and bead width *B* was positive, according to specimen from R1 to R7 in Figure 4. As the increasing of *D_T_*, the *B* was also increasing. While the relevance between welding speed *V_R_* and bead width *B* was negative based on specimen from R8 to R11 in Figure 4, which showed that as the increasing of the welding speed, the bead width was decreasing. The effect of the twin pulse frequency TPF on the bead width *B* was so low based on specimen from R12 to R16 in Figure 4, the relevance was negative and was not obvious, and varying range of the weld width was relatively small. Also, the effects of average welding current *I_av_* and twin pulse current change in percent *I_Δ_* on the bead width *B* were positive, they had similar variation tendencies, respectively based on the specimen from R17 to R21 and from R22 to R26 in Figure 4.

**Figure 5:**
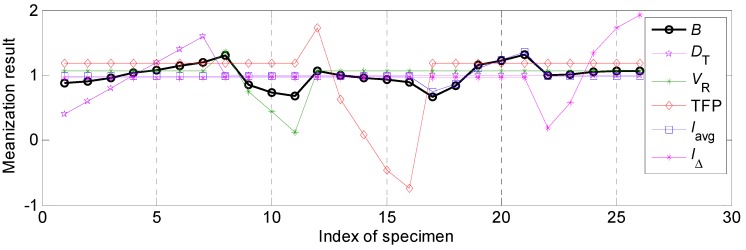
The normalized results of various process parameters and bead width after linear transformation for special parameters.

In general, the detailed relational analysis was conducted under the circumstance that all the relevancies between the data in the analysis element sequence and the objective parameter, which was the bead width in this part, had the same polarity, in other words, all the relevancies should be positive or all the relevancies should be negative. However, in above analysis both positive and negative relevance appeared. The relevancies between welding speed as well as twin pulse frequency, and bead width, were negative. The increasing of welding speed and twin pulse frequency may make the weld width decrease. If all the relational degrees of these five process parameters were calculated follows the *Step* 4, inaccurate results can be obtained, due to the process parameters which had negative relevancies may obtain very small values of relational degree. To obtain accurate and convinced calculation, it was required that making necessary transforming for the special process parameters. This was a necessary operation from qualitative analysis to quantitative analysis during the relational analysis process. The following linear transformation did not affect the final results of relational analysis, and only changed the original negative relevancy to positive relevancy.

(12) $$x_{i}{\left(k\right)}^{'}=2\times \frac{1}{m}\sum_{k=1}^{m}x_{i}^{}\left(k\right)-x_{i}\left(k\right),$$

where $x_{i}{\left(k\right)}^{'}$ is the original element in the analysis element sequence after linear transformation. It can be noticed that the Equation (12) was to change the polarity of the original data, at the same time added a twice of the mean value of the original data. Hence, this operation can only change the polarity, and make the comparison of grey relational degrees and corresponding rank accurate. After linear transformation for the special parameters, which were welding speed and twin pulse frequency in this section, it can be observed from Figure 5 that through transformation using Equation (12), the relevancies between welding speed, twin pulse frequency and the bead width were changed into positive. 

Under the circumstance, all the relevancies were positive and relational coefficients can be calculated, and the corresponding results were shown in Table 5. It can be noticed that the effect of welding speed on the bead width was the highest. During the whole working range, the variation range of the bead width can achieve 4.05 mm, the following influential factor was the average welding current, corresponding variation range was 3.87 mm. The twin pulse current change in percent had a lowest effect on the bead width, the variation range was only 0.44 mm. Apart from the relevancies between welding speed, twin pulse frequency and bead width was negative, the relevance between other process parameters and bead width were positive.

Also, according to Table 5, the influential degrees of different process parameters on the bead width were: *I_av_* > *V_R_* > *D_T_* > TPF > *I*_Δ_, according to their different errors shown in the Table 5; while the grey relational rank of these parameters were: *I_av_* > *V_R_* > *D_T_* > *I*_Δ_ > TPF, which was based on the grey relational degree calculation. 

#### 4.2.2. The Influential Rules of Process Parameters on Bead Height and Corresponding Relational Degree

**Figure 6:**
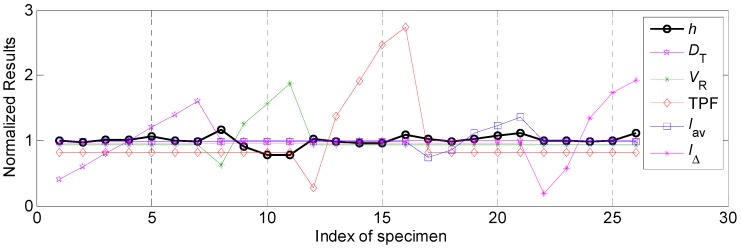
The normalized results of various process parameters and bead height.

The normalized results of various process parameters and penetration *h* were shown in Figure 6. The correspondence between experimental data and process parameters analyses was the same as that in Section 4.2.1. It can be noticed that the relevancies between welding speed, twin pulse frequency and bead height were negative, while relevancies between other process parameters and bead height were positive. 

**Figure 7:**
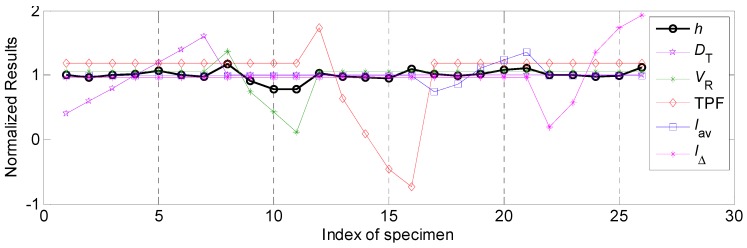
The normalized results of various process parameters and bead height after linear transformation for special parameters.

Similar to preceding section, Equation (12) was employed to make transformation for welding speed and twin pulse frequency, corresponding normalized results after transformation were shown in Figure 7. It can be seen that the bead height had larger variation when the welding speed and average welding current changed than that of other process parameters. 

The influential ranges and relational coefficients of the process parameters were shown in Table 6. It can be noticed that the effects of average welding current and welding speed on the bead height were significantly higher than those of other parameters. During the whole working range, the variation range of the bead height was 0.92 mm. The following influential factor was twin pulse current change in percent, corresponding variation range of the bead height was 0.32 mm. The lowest effect was appeared in the twin pulse relation, the corresponding variation range of the bead height was only 0.22 mm. 

Also, according to Table 6, the influential degrees of different process parameters on the bead height were: *V_R_* > *I*_Δ_ > TPF > *I_av_* > *D_T_*, while the Grey relational rank of these parameters were: *I_av_* > *D_T_* > *V_R_* > *I*_Δ_ > TPF. 

#### 4.2.3. The Influential Rules of Process Parameters on Penetration and Corresponding Relational Degree

**Figure 8:**
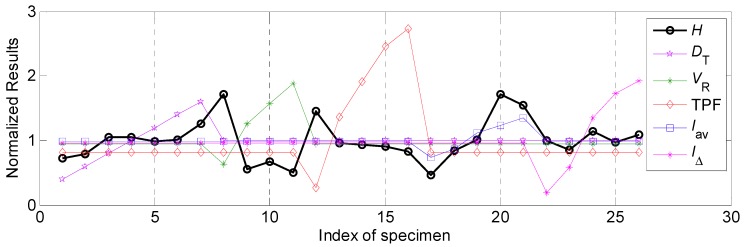
The normalized results of various process parameters and penetration.

The normalized results of various process parameters and penetration *H* were shown in Figure 8. The correspondence between experimental data and process parameters analyses was also the same as that in Section 4.2.1. It can be noticed that the relevancies between welding speed, twin pulse frequency and penetration were negative, while relevancies between other process parameters and penetration were positive.

**Figure 9:**
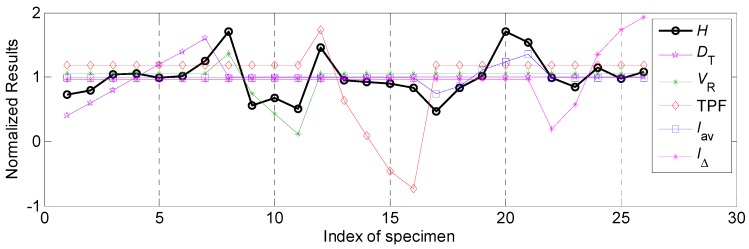
The normalized results of various process parameters and penetration after linear transformation for special parameters.

Equation (12) was also employed to make transformation for the welding speed and twin pulse frequency. Corresponding normalized results after linear transformation were shown in Figure 9. It can be seen that the penetration had larger variation when the welding speed and average welding current changed than that of other process parameters.

The influential ranges and relational coefficients of the process parameters were shown in Table 7. It can be noticed that the effects of average welding current and welding speed on the penetration were significantly higher than those of other parameters. During the whole working range, the variation range of the penetration using different average welding currents was 2.18 mm. The following influential factor was welding speed, corresponding variation range of the penetration was 2.11 mm. The lowest effect was occurred in the twin pulse current change in percent, the variation range of the bead height was only 0.51 mm. The penetration and average welding current were approximately a direct ratio. Too small value of average welding current can induce unstable electrical arc and low penetration, and no penetrated phenomena and slags may appear. Also, the production rate was deteriorated. Increasing of the average welding current can increase the electrical arc force and heat delivery to the base plate, the temperature of the weld bead was so high, which can induce too wide bead width and too deep penetration, as well as burn-through and undercut phenomena might appear, which seriously deteriorate the welding quality. Hence, the selection of welding current was so important and should be proper. 

Also, according to Table 7, the influential degrees of different process parameters on the penetration were: *I_av_* >*V_R_* > TPF > *D_T_* > *I*_Δ_, while the grey relational rank of these parameters were: *I_av_* > *V_R_*> *D_T_* > *I*_Δ_ > TPF.

### 4.3. Grey Relational Analysis of Weld Bead Forming and Comprehensive Effect on the Characteristic Process of the Weld Bead 

In Section 4.2, the grey relational degrees between each process parameter and three key characteristic parameters of the weld bead were obtained by corresponding calculations, as well as the influential variation ranges and relevancies were also provided. Then, the comprehensive effects of each process parameter on the weld bead forming and three characteristic parameters were required to be deeply considered. In this section, the gray relational degrees between weld bead forming characteristic parameter and process parameters, as well as the mean values of gray relational degrees between the process parameters and the key characteristic parameters, which had been calculated in preceding sections, were employed to analyze. 

In this work, weld forming factor was introduced to describe the forming situations of the weld bead. Its definition was a ratio between the bead width (*B*) and penetration (*H*) shown in Figure 2, the mathematical description was as follows:

*φ* = *B*/*H* (13)

where *φ* was the weld forming factor. According to the definition, it can be observed that small weld forming factor meant that the weld bead had small bead width and deep penetration, and more impurities may exist in the middle of the bead, which can induce poor resistance to hot crack appearing. While large weld forming factor meant that the weld bead had large bead width and shallow penetration, which can induce the weld bead was not beautiful and the strength of the weld bead was also deteriorated. Hence, the relation between weld forming factor and the weld bead formation was so close. Therefore, the grey relational degrees between the weld forming factor between the process parameters, can be employed to explore the influential variation range and grey relational degree of various process parameters. 

**Figure 10:**
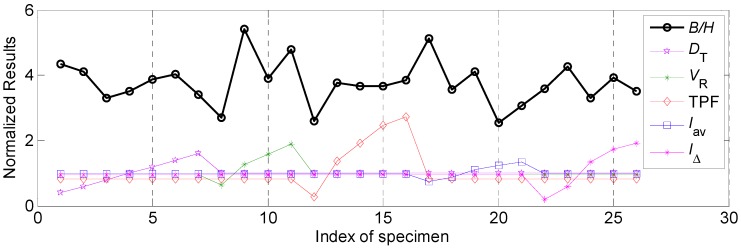
The normalized results of various process parameters and weld forming factor.

Figure 10 showed the normalized results of various process parameters and weld forming factor. The correspondence between experimental data and process parameters analyses was the same as that in in Section 4.2.1. To clearly show the comparative relations, the weld forming factor in Figure 10 was not processed using normalized method. It can be observed from Figure 10 that the values of weld forming factor in this work were between 2 and 5, and no obvious monotonic variations between different process parameters and weld forming factor appearing. Hence, it was no required to make transformation in this part. 

Then the variation range and grey relational degree of the weld forming factor was calculated. During the calculations of grey relational degrees, all the data included weld forming factor after normalized processed was used. Corresponding results were shown in Table 8. It can be seen that the effect of the welding speed on the weld forming factor was the highest, whose variation range was 2.70, the following influential factor was average welding current, and the corresponding variation range was 2.57. In addition, the effects of other three process parameters on the weld forming factor were relatively low, the variation ranges were around 1. 

The grey relational degrees between various process parameters and weld forming factor were ranked as follow: *V_R_*> *I_av_*> *D_T_* > *I*_Δ_ > TPF. In addition, the influential ranges of various process parameters on the weld forming factor were as follows: *V_R_*> *I_av_*> *D_T_* > TPF > *I*_Δ_.

Then the mean values of the grey relational degree of the bead width *B*, bead height *h* and penetration *H* can be calculated, which can effectively complement the pervious analysis and explore the comprehensive effects on the three process parameters, as shown in Table 9.

It can be seen that the corresponding rank were as follows: *I_av_*> *V_R_* > *D_T_* > *I*_Δ_ > TPF.

According to above analyses, it can be noticed that no matter for the weld forming factor, or for the three key characteristic parameters of the weld bead forming, which were bead width *B*, bead height *h* and penetration *H*, grey relational degrees of average welding current *I_av_* and welding speed *V_R_* were larger than that of other three process parameters. It meant that the effects of these two parameters on the weld bead forming were larger. Large value of weld forming factor meant that the weld bead was so wide and the penetration was so shallow, the penetration rate was not enough; while small value of weld forming factor meant that the weld bead was so thin and the penetration was so deep, which might easily induce hot crack. So, the value of weld forming factor cannot be so small. Hence, appropriate welding speed *V_R_* and average welding current *I_av_* should be employed to obtain satisfactory welding performance. The value of welding speed was directly related to the production rate of the welding operation. High welding speed might make all the three key characteristic parameters decrease, so this parameter was negative related to above process objective. This was because that when the welding speed increased, the extern heat effected on the one-unit length of the base plate decreased, which induced the melting metal decreased, and corresponding sizes were also deceased. In addition, the effects of average welding current on the welding quality and production rate were also so high, and this parameter can directly affect the weld bead forming. The relevancies between average welding current and the three key characteristic parameters of the weld bead forming were positive. This was because higher average welding current can make the temperature increase, and melting metal also increased, which induced the penetration increased. Moreover, as the increasing of the welding current, the electrical arc voltage also increased. The increased electrical arc voltage can induce the length of electrical arc increased, then the heating area also increased, and then the bead width also increased. All the relevancies between twin pulse relation and the three key characteristic parameters were positive, and the grey relational grade ranked the third. It had the same influential trend as that of average welding current, but with smaller influential variation range. It might because that increasing of twin pulse relation can make the average welding current increase, though the increasing magnitude was a bit limited. Hence, the corresponding trend was the same as that of average welding current but with smaller influential variation range. The grey relational degrees between twin pulse current change in percent and the three key characteristic parameters were ranked the fourth, and the influential variation range was so small, which meant the effect was relatively low. Finally, the grey relational degrees between twin pulse frequency and the three key characteristic parameters were the lowest, when compared to other process parameters. It meant that the effect of the TPF on the weld forming during DP-GMAW process was not remarkable. According to previous researches and the examination of microstructure [26], the main function of TPF was to obtain beautiful fish scale ripple, and to refine the grain of the weld bead. 

## 5. Conclusions

According to the research, experiments and corresponding analyses in this work, some important conclusions can be drawn as follows:

1. The grey relational analysis method can confirm the influential degrees of various process parameters on the final results, and then instruct the process parameters selection and process optimization, under the circumstance that the number of the specimens was limited and the relations between the elements and final results were not obvious. According to the grey relational analyses, the average welding current and welding speed were the key elements which affected the characteristic parameters of the weld bead, no matter according to the grey relational degree or the influential range, corresponding values of these two process parameters were so large. On the other hand, other process parameters, which were twin pulse relation, twin pulse current change in percent, twin pulse frequency, corresponding relational degrees about the weld forming were relatively low, and the influential ranges were also not remarkable.
2. According to analyses based on the normalized values of various process parameters and three characteristic parameters of the weld bead, the relevancies between twin pulse relation, average welding current and twin pulse current change in percent, and three characteristic parameters of the weld bead, were positive, while the relevancies about the welding speed and twin pulse frequency were negative.
3. As for the weld forming factor, the effects of different process parameters were not monotonous. This was because that the influential ranges of different process parameters on the bead width and penetration were different. Even though, the effects of average welding current and welding speed on the weld bead forming were significantly higher than those of other process parameters.

## Figures and Tables

**Table 1:** Material characteristics of the base plate and welding wire.

Materials	C	Si	Mn	Cr	Ni	S	P	N	Mo
Stainless steel 304	≤0.08	≤1	≤2	18~20	8~10.5	≤0.03	≤0.03	≤0.1	-
Stainless steel 316L	≤0.03	≤ 1	≤2	16~18	10~14	≤0.03	≤0.045	-	2~3

**Table 2:** Experimental program of the selected process parameters.

Index	*D_T_* (%)	*V_R_* (cm/min)	TPF (Hz)	*I_av_* (A)	*I_Δ_* (%)
R1	20%	30 cm/min	1.5 Hz	80 A	25%
R2	30%	30 cm/min	1.5 Hz	80 A	25%
R3	40%	30 cm/min	1.5 Hz	80 A	25%
R4	50%	30 cm/min	1.5 Hz	80 A	25%
R5	60%	30 cm/min	1.5 Hz	80 A	25%
R6	70%	30 cm/min	1.5 Hz	80 A	25%
R7	80%	30 cm/min	1.5 Hz	80 A	25%
R8	50%	20 cm/min	1.5 Hz	80 A	25%
R9	50%	40 cm/min	1.5 Hz	80 A	25%
R10	50%	50 cm/min	1.5 Hz	80 A	25%
R11	50%	60 cm/min	1.5 Hz	80 A	25%
R12	50%	30 cm/min	0.5 Hz	80 A	25%
R13	50%	30 cm/min	2.5 Hz	80 A	25%
R14	50%	30 cm/min	3.5 Hz	80 A	25%
R15	50%	30 cm/min	4.5 Hz	80 A	25%
R16	50%	30 cm/min	5 Hz	80 A	25%
R17	50%	30 cm/min	1.5 Hz	60 A	25%
R18	50%	30 cm/min	1.5 Hz	70 A	25%
R19	50%	30 cm/min	1.5 Hz	90 A	25%
R20	50%	30 cm/min	1.5 Hz	100 A	25%
R21	50%	30 cm/min	1.5 Hz	110 A	25%
R22	50%	30 cm/min	1.5 Hz	80 A	5%
R23	50%	30 cm/min	1.5 Hz	80 A	15%
R24	50%	30 cm/min	1.5 Hz	80 A	35%
R25	50%	30 cm/min	1.5 Hz	80 A	45%
R26	50%	30 cm/min	1.5 Hz	80 A	50%

**Table 3:** The appearances and cross sections of weld beads of R1–R7.

No.	Weld Bead	
	Appearance	Cross Section
R1	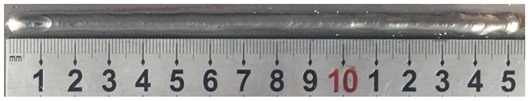	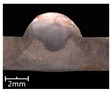
R2	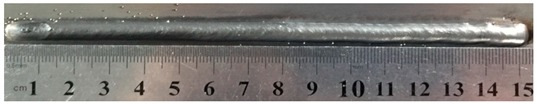	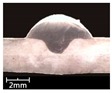
R3	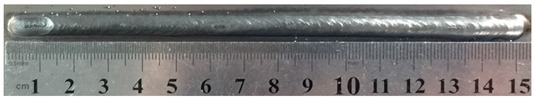	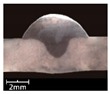
R4	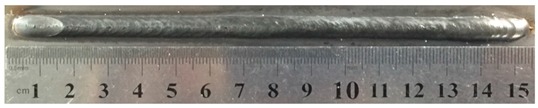	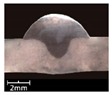
R5	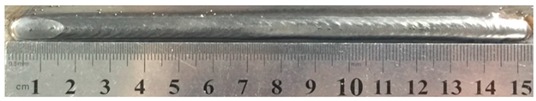	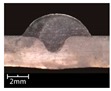
R6	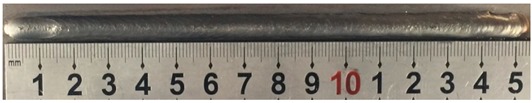	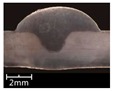
R7	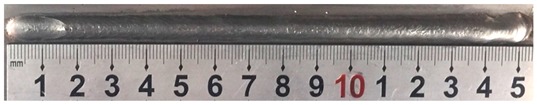	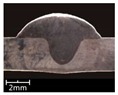

**Table 4:** Measurement of the weld bead (\mm).

**No.**	**1**	**2**	**3**	**4**	**5**	**6**	**7**	**8**	**9**	**10**	**11**	**12**	**13**
*B*	5.51	5.68	6.02	6.46	6.71	7.16	7.5	8.13	5.31	4.61	4.26	6.63	6.29
*h*	2.35	2.27	2.36	2.37	2.49	2.35	2.3	2.74	2.13	1.84	1.82	2.4	2.3
*H*	1.27	1.38	1.83	1.84	1.73	1.78	2.2	3.17	0.98	1.18	0.89	2.55	1.67
**No.**	**14**	**15**	**16**	**17**	**18**	**19**	**20**	**21**	**22**	**23**	**24**	**25**	**26**
*B*	5.99	5.81	5.58	4.2	5.24	7.28	7.65	8.25	6.26	6.35	6.59	6.7	6.68
*h*	2.26	2.24	2.55	2.39	2.32	2.39	2.53	2.6	2.35	2.34	2.3	2.33	2.62
*H*	1.63	1.58	1.45	0.82	1.47	1.77	3.00	2.7	1.74	1.49	2.00	1.71	1.9

**Table 5:** Grey relational analysis about the bead width *B.*

	Process Parameter (Influential Factor)				
Item	*D_T_*	*V_R_*	TPF	*I_av_*	*I_Δ_*
Max (mm)	7.50	8.13	6.63	8.25	6.70
Min (mm)	5.51	4.26	5.58	4.20	6.26
Error (mm)	1.99	3.87	1.05	4.05	0.44
Grey relational degree	0.85	0.89	0.77	0.91	0.82
Relevance	Positive	Negative	Negative	Positive	Positive
Grey relational rank	3	2	5	1	4

**Table 6:** Grey relational analysis about the bead height *h*.

	Process Parameter (Influential Factor)				
Item	*D_T_*	*V_R_*	TPF	*I_av_*	*I_Δ_*
Max (mm)	2.49	2.74	2.55	2.60	2.62
Min (mm)	2.27	1.82	2.24	2.32	2.30
Error (mm)	0.22	0.92	0.31	0.28	0.32
Grey relational degree	0.887	0.874	0.770	0.925	0.867
Relevance	Positive	Negative	Negative	Positive	Positive
Grey relational rank	2	3	5	1	4

**Table 7:** Grey relational analysis about the penetration *H.*

	Process Parameter (Influential Factor)				
Item	*D_T_*	*V_R_*	TPF	*I_av_*	*I_Δ_*
Max (mm)	2.20	3.00	2.55	3.00	2.00
Min (mm)	2.27	0.89	1.45	0.82	1.49
Error (mm)	0.93	2.11	1.10	2.18	0.51
Grey relational degree	0.80	0.83	0.73	0.85	0.78
Relevance	Positive	Negative	Negative	Positive	Positive
Grey relational rank	3	2	5	1	4

**Table 8:** Grey relational analysis of weld forming factor and process parameters.

	Process Parameter (Influential Factor)				
Item (for Weld Forming Factor)	*D_T_*	*V_R_*	TPF	*I_av_*	*I_Δ_*
Max	4.34	5.41	3.85	5.12	4.26
Min	3.29	2.71	2.60	2.55	3.30
Error	1.05	2.70	1.25	2.57	0.97
Grey relational degree	0.83	0.87	0.75	0.86	0.81
Grey relational rank	3	1	5	2	4

**Table 9:** Comprehensive grey relational analysis of weld bead forming and process parameters.

	Process Parameter (Influential Factor)				
Item	*D_T_*	*V_R_*	TPF	*I_av_*	*I_Δ_*
Grey relational degree related to bead width *B*	0.85	0.89	0.77	0.91	0.82
Grey relational degree related to bead height *h*	0.89	0.87	0.77	0.93	0.87
Grey relational degree related to penetration *H*	0.80	0.83	0.73	0.85	0.78
Mean values of the comprehensive grey relational degree	0.85	0.86	0.76	0.90	0.82
Comprehensive grey relational rank	3	2	5	1	4
